# Transplant-Related Mortality Following Allogeneic Hematopoeitic Stem Cell Transplantation for Pediatric Acute Lymphoblastic Leukemia: 25-Year Retrospective Review

**DOI:** 10.1002/pbc.24559

**Published:** 2013-06-03

**Authors:** Marion K Mateos, Tracey A O’Brien, Cecilia Oswald, Melissa Gabriel, David S Ziegler, Richard J Cohn, Susan J Russell, Draga Barbaric, Glenn M Marshall, Toby N Trahair

**Affiliations:** 1 Kids Cancer Centre, Sydney Children’s HospitalRandwick, NSW, Australia; 2 School of Women and Children’s Health, University of New South WalesNSW, Australia; 3 The Children’s HospitalWestmead, NSW, Australia

**Keywords:** hematopoeitic stem cell transplant, lymphoblastic leukemia, outcomes, pediatric acute survival, transplant-related mortality

## Abstract

**Background:**

Over the last 25 years, donor source, conditioning, graft-versus-host disease prevention and supportive care for children undergoing hematopoeitic stem cell transplantation (HSCT) have changed dramatically. HSCT indications for acute lymphoblastic leukemia (ALL) now include high-risk patients in first and subsequent remission. There is a large burden of infectious and pre-HSCT morbidities, due to myelosuppressive therapy required for remission induction. We hypothesized that, despite these trends, overall survival (OS) had increased.

**Procedure:**

A retrospective audit of allogeneic pediatric HSCT for ALL was performed in our institution over 25 years. Outcomes for 136 HSCTs were analyzed in three consecutive 8-year periods (Period 1: 1/1/1984–31/8/1992, Period 2: 1/9/1992–30/4/2001, Period 3: 1/5/2001–31/12/2009).

**Results:**

Despite a significant increase in unrelated donor HSCT, event-free and OS over 25 years improved significantly. (EFS 31.6–64.8%, *P* = 0.0027; OS 41.8–78.9%, *P* < 0.0001) Concurrently, TRM dropped from 33% to 5% (*P* = 0.0004) whilst relapse rate was static (*P* = 0.07). TRM reduced significantly for matched sibling and unrelated cord blood transplantation (UCT) in Period 3 compared with earlier periods (*P* = 0.036, *P* = 0.0098, respectively). Factors leading to improved survival in patients undergoing UCT include better matching, higher total nucleated cell doses, and significantly faster neutrophil engraftment. Length of initial HSCT admission was similar over time.

**Conclusion:**

EFS and OS have increased significantly despite heightened HSCT complexity. This survival gain was due to TRM reduction. Contemporary patients have benefited from refined donor selection and improved supportive care. Overall rates of leukemic relapse post-HSCT are unchanged, and remain the focus for improvement.

## INTRODUCTION

Refinements in pediatric acute lymphoblastic leukemia (ALL) treatment from collaborative clinical trials have achieved cure rates of 85% [1] and 5-year event-free survival (EFS) of 80% [2]. Relapsed leukemia remains the commonest cause of pediatric cancer death.

Allogeneic hematopoeitic stem cell transplantation (HSCT) in pediatric ALL is considered using clinical and laboratory features such as length of first remission in relapsed patients and minimal residual disease (MRD) in first remission [3]. MRD monitoring has facilitated risk stratification and treatment selection [3–8]. Allogeneic HSCT indications are evolving [9,10], as survival with chemotherapy-only treatment improves [3,4,9,11].

Donor sources for HSCT have changed over the last 25 years. A matched sibling donor (MSD) was historically preferred over unrelated donors, due to perceived transplant-related mortality (TRM) risk [12–14]. Limited MSD availability has prompted use of alternate donors, including family-related donors (FRD), matched unrelated donors (MUD), and matched unrelated cord blood transplants (UCT). UCT is increasingly used, due to factors including wider range of compatible donors and ease of availability [15]. In childhood ALL, alternate donor HSCTs comprise 61% of allografts (1999–2002) [14] whereas prior to 1996, MSD accounted for 70% of HSCT [14]. UCT now accounts for one-third of HSCT for acute leukemia [16].

With the advent of alternate donor HSCT, TRM risk has increased [17]. Audits of adult patients demonstrate this [17,18]. In addition, relapsed patients often require greater myelosuppression to achieve pre-HSCT remission [16,18–20], with consequences of increased pre-HSCT comorbidities and infective burden. Despite a higher recent predicted TRM, one study involving adults showed significant improvement in survival and reduction in overall mortality, including non-relapse and relapse mortality [18]. TRM figures for pediatric HSCT, including ALL patients, are 5–10% for MSD [3,21,22], 8–24% for MUD [3,6,21,22], and over 20% for mismatched donors [3,21,23]. Earlier TRM figures for unrelated donor HSCT in pediatric ALL were 16–42% [22,24,25].

Although there are general trends of improved overall survival (OS) and reduced TRM [17,26,27] following HSCT, few studies have discussed temporal trends in pediatric ALL HSCT [16,22,25]. We analyzed our experience with pediatric HSCT between 1984 and 2009 in ALL. We hypothesized that OS in our allogeneic HSCT cohort had improved, despite increase in unrelated donor HSCT.

## PATIENTS AND METHODS

### Data Collection

A retrospective review of 136 consecutive allogeneic HSCT for ALL at Sydney Children’s Hospital was conducted. Data from 1/1/1984 to 31/12/2009 were analyzed from the Cord & Marrow Transplant database at Sydney Children’s Hospital. Comprehensive data were collected prospectively in the Transplant database with internal and external audits to assess data accuracy. The data set was censored at 31/12/2010. Data for survivors were censored at date of last follow-up. This study received institutional ethics committee approval.

The study period was divided into three time frames (Period 1: 1/1/1984–31/8/1992, Period 2: 1/9/1992–30/4/2001, Period 3: 1/5/2001–31/12/2009) and HSCT outcomes analyzed. Time frames reflected changes in choice of alternate donors, with predominant use of FRD in Period 1, MUD in Period 2 and UCT in Period 3. Similar numbers of HSCT were performed in each period (Table I).

**TABLE I:** Characteristics of Each Period

	Period 1 1/1/1984–31/8/1992	Period 2 1/9/1992–30/4/2001	Period 3 1/5/2001–31/12/2009	*P-*value
Number of HSCT^a^	41	48	47	
Number of patients	40	46	44	
Baseline characteristics				
Males	30 (73.2%)	35 (72.9%)	31 (66%)	0.7
Median age (months) at HSCT (range)	117 (9–235)	97 (10–206)	108 (17–221)	0.48
Median time (months) from diagnosis to HSCT (range)	36 (4–114)	34 (5–122)	23 (4–80)	<0.01
Remission status				
CR1	4 (9.8%)	4 (8.3%)	15 (31.9%)	0.003
CR2	23 (56.1%)	35 (72.9%)	25 (53.2%)	0.108
CR3	12 (29.3%)	7 (14.6%)	5 (10.6%)	0.058
CR4	1 (2.4%)	1 (2.1%)	0	0.59
Not documented	0	0	2 (4.3%)	N/A^h^
Persistent disease	1 (2.4%)	1 (2.1%)	0	N/A^h^
Radiotherapy administered prior to HSCT conditioning	12 (29.3%)	11 (22.9%)	1 (2.13%)	<0.01
Graft type				
MSD^b^	27 (65.9%)	20 (41.7%)	15 (31.9%)	0.02
UCT^c^	0	8 (16.7%)	21 (44.7%)	<0.01
MUD^d^	2 (4.9%)	12 (25%)	10 (21.3%)	0.033
FRD^e^ (Haplo/MMSD^f^)	12 (29.3%)	8 (16.7%)	1 (2.1%)	0.002
T cell depletion	8 (19.51%)	16 (33.3%)	10 (21.3%)	0.25
Conditioning type				
TBI-based conditioning	15 (36.6%)	31 (64.6%)	45 (95.7%)	<0.01
Chemotherapy alone	26 (63.4%)	17 (35.4%)	2 (2.1%)	<0.01
CMV status (donor/recipient)				
D−/R−g	16 (39%)	14 (29.2%)	25 (53.2%)	0.06
D−/R+	3 (7.3%)	8 (16.7%)	11 (23.4%)	0.125
D+/R+	10 (24.4%)	16 (33.3%)	5 (10.6%)	0.03
D+/R−	9 (22%)	9 (18.8%)	6 (12.8%)	0.52
Not tested	3 (7.3%)	1 (2.1%)	0	N/A^h^

a Hematopoeitic stem cell transplant

b Matched sibling donor

c Umbilical cord blood transplant

d Matched unrelated donor

e Family-related donor

f Mismatched sibling donor

g D−/R− denotes that pre-transplant CMV status is negative for donor, negative for recipient

h *P*-value not generated as numbers for analysis are too small.

Patients who received HSCT in CR1 fulfilled high-risk or very high-risk features on ALL protocols used. Risk stratification was based on Berlin–Frankfurt–Munster (BFM) criteria, and incorporated MRD criteria in Period 3.

Conditioning regimens varied according to transplant period (Table I). There was a significant increase in TBI use (*P* < 0.01), with predominantly TBI-based conditioning in Period 3. Four reduced-intensity transplants were performed in Period 3, one upfront and three for post-HSCT relapse. In Period 3, 12.5% of patients who received TBI-based myeloablative conditioning also received a CNS boost. No CNS boost was administered in earlier periods. TBI was generally avoided in those aged under 2 years at time of HSCT. Overall, 89% of chemotherapy-alone conditioning contained cyclophosphamide and busulphan. In Period 1, a regimen of busulphan, cyclosphosphamide, and melphalan was used in 74% of HSCT episodes. The combination of busulphan, cyclosphamide, and etoposide was used in 53% of HSCT in Period 2. Busulphan levels were not obtained. Anti-thymocyte globulin (Pfizer) was used for UCT and MUD HSCT at 12 mg/kg/dose for three doses, including T-cell-depleted MUD HSCT. Anti-thymocyte globulin was prophylaxis for rejection and graft-versus-host disease (GVHD).

Graft characteristics are shown in Table I. The majority of patients who underwent MUD HSCT received bone marrow. Tissue typing and HLA matching were performed by the Australian Bone Marrow Donor Registry (ABMDR) using established international methods [28,29]. High-resolution typing for DRB1 has been used since the late 1990s and high-resolution typing for Class 1 allele (HLA-ABC) testing has been routine in recent years [22]. UCT were 4/6 matches or better (Table III). FRD were mostly one to two antigen mismatches, and included mismatched siblings, parents or relatives. In Period 1, one FRD was a haploidentical parent.

Supportive care practices in our unit varied prior to 2004. We used graft support with colony-stimulating factor from 1991 for unrelated donor HSCT. Cyclosporine was the consistent backbone of GVHD prophylaxis. For patients receiving MSD HSCT after 2004, cyclosporine and methotrexate were given as GVHD prophylaxis. Patients transplanted using single UCT prior to 2004 received cyclosporine, methylprednisone, and methotrexate for GVHD prophylaxis, whereas only cyclosporine and methylprednisone were used after 2004. Cyclosporine and mycophenolate were used for GVHD prophylaxis in patients receiving double UCT. For patients undergoing MUD HSCT prior to 2000, cyclosporine and red cell E-rosette were used for GVHD prophylaxis, whilst cyclosporine and CD34 selection together were used after 2000. Penicillin prophylaxis was used in Period 3 for patients with GVHD.

In Period 3, patients who were CMV IgG positive received ganciclovir therapy (pre-HSCT and post-engraftment) and weekly CMV immunoglobulin to Day +100. Qualitative CMV PCR monitoring was routine from 2004 and quantitative PCR from 2008. PJP (*Pneumocystis jirovecii pneumonia*) prophylaxis included routine pentamidine (since 2004) and cotrimoxazole post-engraftment. Patients who were HSV IgG positive received aciclovir. Additional changes from 2004 were routine antifungal prophylaxis—fluconazole for standard risk and liposomal amphotericin or voriconazole for patients at high risk of fungal disease; routine ursodeoxycholic acid for veno-occlusive disease prophylaxis [30], with additional prophylactic defibrotide for high-risk patients [31]. Features indicating higher VOD risk included heavily pre-treated patients, prior HSCT, underlying liver dysfunction, hepatic iron overload, and causative drugs [32].

### Statistical Analysis and Definitions

OS, EFS, leukemia-free survival (LFS), TRM, and cumulative incidence of relapse were analyzed from time of HSCT. Kaplan–Meier survival curves were constructed [18,33] and sub-analyses performed to compare survival according to graft type, period of HSCT, and remission status. Survival outcomes for Periods 1 and 2 were not statistically different and were combined for analysis. Each HSCT episode was analyzed separately for survival and TRM.

An event was defined as relapse, TRM, death from any cause or second malignancy. TRM was defined as death due to complication (other than relapse) following HSCT. LFS was defined as alive, without evidence of leukemia, or leukemia-free death. A second malignancy was defined as malignancy other than the primary leukemia, occurring post-HSCT.

Viral, bacterial and fungal infections were analyzed as recorded on the Transplant database. Neutrophil engraftment was defined as the first of three consecutive days of ANC ≥0.5 × 10^9^/L. Platelet engraftment was defined as unsupported platelet count >20 × 10^9^/L. Acute GVHD (aGVHD) was graded according to the Glucksberg severity scale [34]. Chronic GVHD was GVHD occurring after Day +100 [34].

Statistical analysis was performed using PRISM (Prism 5, GraphPad Software, Inc. 2005–2010). Two-sided *P* values (Mantel–Cox Log Rank) were significant if below 0.05 and 95% confidence intervals used. PASW Version 18 software was used for variance analysis (ANOVA, *t* tests, Kruskall Wallis chi-squared tests, Cox-regression).

## RESULTS

### Patient Population

An overview of HSCT characteristics and outcomes is shown in Tables I and II, respectively. The groups had similar pre-HSCT characteristics, apart from remission status and time from diagnosis to HSCT. There was a significant increase in patients transplanted in CR1 in Period 3 (*P* = 0.003). This increase reflected use of HSCT for patients with high-risk disease based on MRD. The time from diagnosis to transplant was shorter in Period 3 compared to earlier periods (*P* < 0.01). There was no difference in time from diagnosis to HSCT for patients transplanted in ≥CR2 over 25 years (*P* = 0.07). There was no increase in patients being transplanted with persistent disease.

**TABLE II:** Outcomes

	Period 1^b^	Period 2^c^	Period 3^d^	
Number of HSCT^a^	41	48	47	*P*-value^e^
5-Year OS (%)	35.70%	46.80%	78.90%	<0.0001
Median survival (months) post-HSCT in months (range)	19 (1–312)	20 (0–198)	29 (2–108)	0.04
TRM	13 (31.7%)	15 (31.3%)	2 (4.3%)	0.0004
Relapses post-HSCT	16 (39%)	16 (33.3%)	12 (25.5%)	0.207
Median time (months) to relapse (range)	7 (3–47)	6 (1–42)	15 (4–30)	0.61
Second malignancy	1 (2.4%)	2 (4.2%)	1 (2.1%)	0.69
5-Year LFS^f^	49.60%	56.60%	68.20%	0.07
5-Year EFS	31.60%	40.40%	64.80%	0.0005
Acute GVHD				
Grade 2–4	16 (39.0%)	11 (22.95%)	18 (38.3%)	0.35
Grade 3–4	10 (24.4%)	7 (14.6%)	9 (19.1%)	0.99
Grade 4	3 (7.3%)	3 (6.3%)	3 (6.4%)	0.94
Chronic GVHD	5 (12.2%)	5 (10.4%)	6 (12.8%)	0.79
Engraftment (days)				
Median neutrophil engraftment (range)	17 (8–28)	20 (10–42)	18 (6–35)	0.68
Median platelet engraftment (range)	22 (11–75)	26 (12–95)	29 (15–91)	0.30
Hospital inpatient days (range)	30 (16–137)	30 (14–87)	34 (25–80)	0.92

a Haematopoietic stem cell transplant

b Period 1: 1/1/1984–31/8/1992

c Period 2: 1/9/1992–30/4/2001

d Period 3:1/5/2001–31/12/2009

e *P* value is for Period 1 and 2 combined as compared to Period 3

f Leukemia-free survival.

Overall median follow-up time for survivors was 75 months (range 4–312 months). Median follow-up for Period 1 was 191 months (range 15–312 months), Period 2 was 133 months (58–198 months), Period 3 was 45 months (4–108 months). Those who died had a median survival of 9 months (range 1–97 months) in Period 1, 4 months (range 0–45 months) in Period 2, and 21 months (range 2–27 months) for Period 3.

### HSCT Characteristics

There were 41 allogeneic HSCT for 40 patients in Period 1. One patient received a second allogeneic HSCT, following relapse post-HSCT. There were 48 HSCT episodes for 46 patients in Period 2. This included two patients who received a second allogeneic HSCT, one for graft failure and the other for relapse post-HSCT. In Period 3, there were 47 HSCT episodes and 44 patients. Subsequent transplants in Period 3 were for relapse post-HSCT. Where a patient received a subsequent HSCT, this was performed in the same period as first HSCT. There were three graft failures, all in patients receiving FRD HSCT (one in Period 1, two in Period 2).

Donor selection changed over time with significant decrease in MSD (*P* = 0.02) and FRD grafts (*P* = 0.002), and significant increase in UCT (*P* < 0.01) and MUD HSCT (*P* = 0.033). In Period 3, mostly UCT or MSD HSCT were performed (Table I). Four double-cord UCT were performed, all in Period 3 (Table III). There were fewer CMV D+/R+ pairs over time (*P* = 0.03) and a trend towards more CMV D−/R− pairs in Period 3 (Table I).

**TABLE III:** UCT Matching, TNC, and Engraftment

	Period 1^a^	Period 2^b^	Period 3^c^	Total	
Total allogeneic transplants	41	48	47	136	*P*-value
UCT^d^	0	8	21	29	<0.001
4/6 match		4 (50%)	2 (10%)	6 (20.7%)	0.028
5/6 match		2 (25%)	11 (52.4%)	13 (44.8%)	0.23
6/6 match		2 (25%)	4 (19%)	6 (20.7%)	0.75
Double cord transplant^e^		0	4 (19%) (3 × 5/6 match, 1 × 4/6)	4 (13.8%)	
Median TNC^f^ (range)		3.35 (0.6–5.0)	4.7 (0.5–8.4)	4.7 (0.5–8.4)	0.08
Median neutrophil engraftment (days, range)		25.5 (18–34)	16 (6–33)	20 (6–34)	0.014
Median platelet engraftment (days, range)		60.5 (19–84)	40.5 (23–91)	46 (19–91)	0.64
Median hospital days (range)		41 (29–87)	45 (29–80)	42 (29–87)	0.16

a Period 1: 1/1/1984–31/8/1992

b Period 2: 1/9/1992–30/4/2001

c Period 3:1/5/2001–31/12/2009

d Umbilical cord blood transplant

e Matching for double cord transplants was according to the least matched cord

f Total nucleated cells × 10^7^/kg.

There was no difference in median neutrophil and platelet engraftment between periods for the entire group (Table II). Graft subanalysis showed median neutrophil engraftment for UCT in Period 3 was significantly faster than Period 2 (Table III). UCT matching improved over time, with a greater percentage of ≥5/6 matched cords between Period 2 and 3 (Table III). In Period 3 UCT, there was a trend towards higher median total nucleated cell doses, although numbers are small (Table III).

There was no significant difference in speed to engraftment for other grafts over time (Table IV). For the 25-year period, median platelet engraftment was significantly longer for UCT (46 days, range 19–91 days) compared to other grafts (*P* < 0.001). Engraftment times are listed in Table III (UCT) and Table IV (other grafts).

**TABLE IV:** Infections (All Grafts) and Engraftment (Other Than Cords)

	Period 1^b^	Period 2^c^	Period 3^d^	Total	
Total HSCT^a^	41	48	47	136	*P-*value
Confirmed infections^e^ (total number, % of HSCT)					
Bacterial	13 (31.7%)	15 (31.3%)	30 (63.8%)	58 (42.7%)	0.001
Viral (except for CMV)	11 (26.8%)	8 (16.7%)	19 (40.4%)	38 (27.9%)	0.035
CMV	5 (12.2%)	7 (14.5%)	7 (15%)	19 (14%)	0.927
Fungal	3 (7.3%)	6 (12.5%)	8 (17%)	17 (12.5%)	0.395
PJP^f^	0	3 (6.3%)	1 (2.1%)	4 (2.9%)	0.206

Outcomes by Graft type (days)	Period 1	Period 2	Period 3	Total	*P*-value
MSD^g^	n = 27	n = 20	n = 15	n = 62	
Median neutrophil engraftment (range)	17 (8–28)	20 (13–42)	19 (12–35)	19 (8–42)	0.14
Median platelet engraftment (range)	21 (12–75)	24 (15–90)	20.5 (17–34)	21 (12–90)	0.63
Median hospital days (range)	27 (14–147)	27 (16–147)	27 (14–87)	27 (14–147)	0.969
FRD^h^	n = 12	n = 8	n = 1	n = 21	
Median neutrophil engraftment (range)	16 (8–28)	15 (13–22)	N/A	16 (8–28)	1
Median platelet engraftment (range)	26.5(21–56)	25 (24–33)	N/A	25.5 (17–56)	1
Median hospital days (range)	62 (19–93)	30 (23–78)	N/A	38 (19–93)	0.052
MUD^i^	n = 2	n = 12	n = 10	n = 24	
Median neutrophil engraftment (range)	13.5 (11–16)	20(10–25)	12.5(10–28)	15.5 (10–28)	0.231
Median platelet engraftment (range)	11.5 (11–12)	23 (12–95)	17 (15–34)	19 (11–95)	0.22
Median hospital days (range)	40 (33–46)	30 (21–67)	34 (25–48)	33 (21–67)	0.45

a Hematopoeitic stem cell transplant

b Period 1: 1/1/1984–31/8/1992

c Period 2: 1/9/1992–30/4/2001

d Period 3:1/5/2001–31/12/2009

e Several infections were often documented in the one patient

f *Pneumocystis jirovecii* pneumonia

g Matched sibling donor

h Family-related donor

i Matched unrelated donor.

### Clinical Outcomes

#### Survival for 25-year period

Overall, 5-year EFS was 45.8%, 5-year OS 53.9%, and 5-year LFS 58.8% (Supplementary Fig 1). TRM was 22.1%. Corresponding survival figures according to HSCT period are listed in Table II. Survival outcomes over 25 years, stratified for remission status, are depicted in Supplementary Fig 1. There was no significant difference in 5-year OS between patients transplanted in CR1 and those transplanted in ≥CR2 (66% vs. 51.2%, *P* = 0.25). TRM for patients in CR1 was not significantly different over 25 years compared to ≥CR2 (9% vs. 26.2%, *P* = 0.14).

#### Survival

Significant improvements in survival were observed when Periods 1 and 2 combined (1/1/1984–30/4/2001) were compared to Period 3 (1/5/2001–31/12/2009).

#### EFS

5-year EFS improved significantly over time, from 36.5% (Periods 1 and 2) to 64.8% (Period 3; *P* = 0.0005). When stratified for remission status, 5-year EFS significantly improved for patients in ≥CR2 over time, increasing from 35.9% to 60.6% (*P* = 0.0093). There was a trend towards improved EFS for patients transplanted in CR2 (*P* = 0.0598).

#### OS

**Figure 1:**
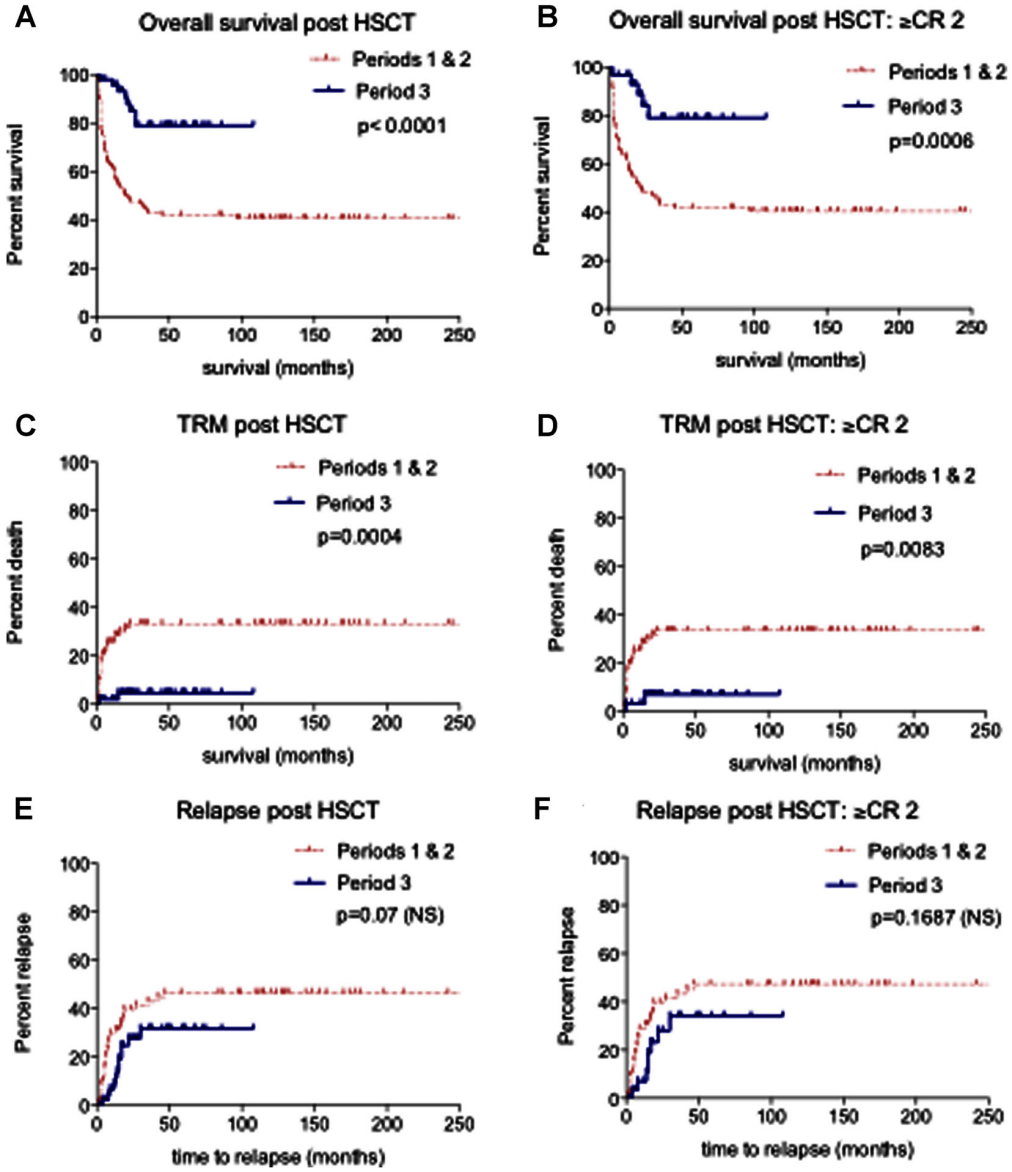
Significant recent improvement in survival and reduced TRM. Periods 1 and 2: 1/1/1984–30/4/2001 (n = 89), Period 3: 1/5/2001–31/12/2009 (n = 47). A and B: Overall survival post HSCT for Periods 1 and 2 compared to Period 3, for all patients (A) and for patients in ≥CR2 (B); C and D: TRM post HSCT for Period 1 and 2 compared to Period 3, for all patients (C) and for patients in ≥CR2 (D); E and F: Relapse post HSCT for Period 1 and 2 compared to Period 3, for all patients (E) and for patients in ≥CR2 (F).

There was a significant increase in 5-year OS from 41.8% (Periods 1 and 2) to 79% (Period 3; *P* < 0.0001). This improvement remained when stratified for remission status. 5-year OS for patients transplanted in CR2 increased from 42.7% (Periods 1 and 2) to 75.4% (Period 3; *P* = 0.0052) and from 41.7% to 79%, respectively for ≥CR2 (*P* = 0.0006; Fig 1).

#### Survival for graft type over 25-year period

Over 25 years, 5-year OS was 53.4% for MSD, 42.9% for FRD, 50.6% for MUD, and 68.4% for UCT. TRM was highest for FRD (39.2%) and ranged between 15% and 25% for other graft types.

#### Graft survival per period

**Figure 2:**
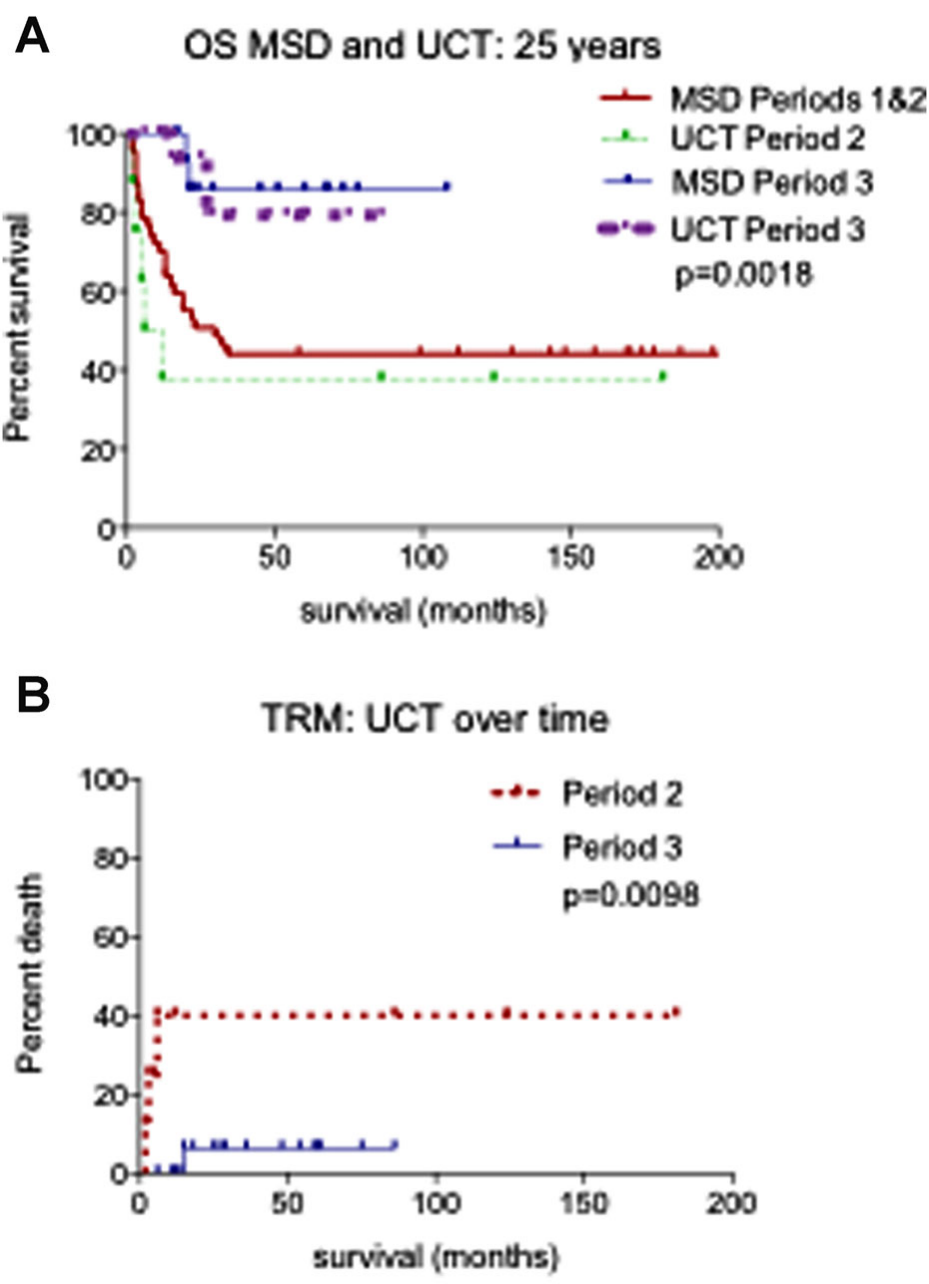
Significant recent survival benefit for MSD and UCT. Periods 1 and 2: 1/1/1984–30/4/2001, Period 3: 1/5/2001–31/12/2009. MSD (matched sibling donor), UCT (umbilical cord blood transplant). A: Overall survival for MSD and UCT cohorts, for Periods 1 and 2 compared to Period 3; B: TRM post UCT for Period 2 compared to Period 3.

There was a significant improvement in 5-year OS for patients receiving MSD and UCT over time (Fig 2). Five-year OS for MSD in Periods 1 and 2 was 44.1% versus 85.7% in Period 3 (*P* = 0.0093). Five-year OS for UCT was 37.5% for Period 2 and 79.3% in Period 3 (*P* = 0.0030). Significant TRM reduction was seen only in MSD and UCT groups. TRM for patients undergoing MSD HSCT for Periods 1 and 2 was 26.9%, and 0% for Period 3 (*P* = 0.036). TRM for patients undergoing UCT in Period 2 was 40% and 6.25% for Period 3 (*P* = 0.0098). In Period 3, there was no significant difference in OS (*P* = 0.80) and TRM (*P* = 0.3329) between recipients of MSD and UCT. Comparative 5-year OS for MUD HSCT was 41% for Periods 1 and 2 and 65.6% for Period 3 (*P* = 0.17).

#### TRM

TRM significantly decreased from 33% (Periods 1 and 2) to 5% (Period 3; *P* = 0.0004). TRM for patients transplanted in CR2 dropped from 31.2% (Periods 1 and 2) to 9.3% for Period 3 (*P* = 0.0417) and for ≥CR2 from 33.6% to 7.3% (*P* = 0.0083) (Fig 1).

#### TRM causes

Deaths from interstitial pneumonitis (IP), infection and GVHD decreased over time. Causes of TRM in Periods 1 and 2 were predominantly IP and GVHD. In Period 1, there were 15 cases of IP, including two CMV and 13 cases with no organism identified (idiopathic). Of these, seven patients died, including both patients with documented CMV. Period 2 also had 15 cases of IP, with five CMV, three PJP, and seven idiopathic. Of these, 10 patients died, including four CMV and three patients with PJP and three idiopathic cases. One patient died from IP in Period 3 (PJP).

GVHD claimed four patients in Period 1 and two patients in Period 2. In Period 1, three patients had multi-organ failure, compared to none in later periods. There were only two TRM deaths in Period 3. There were no deaths from VOD. Where TRM was multi-factorial (five patients in Period 1, three patients in Period 2 and one in Period 3), the main factors were GVHD and infection.

#### Relapse

The 5-year cumulative incidence of relapse did not vary over time (Periods 1 and 2 vs. Period 3: 46.5% vs. 31.6%, *P* = 0.07) nor when stratified for remission status. Five-year cumulative incidence of relapse for patients transplanted in CR2 was 50.1% in Periods 1 and 2 compared to 40.8% in Period 3 (*P* = 0.3229) and for ≥CR2 was 47.2% and 34.1%, respectively (*P* = 0.1687; Fig 1). There was no difference in LFS stratified by graft type (Supplementary Appendix I).

#### HSCT-related complications

There were more bacterial and viral (non-CMV) infections post-HSCT in Period 3 (*P* = 0.001, *P* = 0.035, respectively; Table IV). CMV and fungal infection rates were unchanged at 15% and 17%, respectively in Period 3 (Table IV).

Despite increased use of unrelated donor HSCT, there was no overall increase in GVHD (Table II). Following UCT, there was significantly more Grade 2–4 aGVHD compared to MUD (*P* = 0.018) and MSD HSCT (*P* = 0.046). Incidence of Grade 3–4 aGVHD for UCT was 24.1%, and Grade 2–4 was 51.7%. Equivalent aGVHD figures for other grafts were: MUD Grade 3–4: 12.5%, Grade 2–4: 20.89%; MSD 17.7%, 29%; FRD 23.8%, 33.3%, respectively. Chronic GVHD rates were similar over time (Table II).

#### Length of stay

There was no difference in length of hospital stay over periods (Tables II and III) or between graft types (*P* = 0.54).

## DISCUSSION

Our data show significant improvement over time in EFS and OS post-HSCT for pediatric ALL, despite significant increase in unrelated donor HSCT. Rates of relapse and major HSCT-related complications, such as severe GVHD, remained steady. The major contributor to improved survival was significant TRM reduction.

We have shown greatest survival and TRM improvements for patients undergoing MSD and UCT. UCT TRM and OS trends over time have not been the focus of previous papers [16,22,35,36]. We observed a significant survival advantage in Period 3 UCT. There are several contributors to this, including better graft selection, with significantly smaller degree of mismatch and trend towards a higher median TNC. This resulted in significantly faster median neutrophil engraftment for Period 3 UCT. Survival benefits of choosing better matched UCT units, with TNC >3 × 10^7^/kg are documented [35]. Total nucleated cell dose alone did not account for the survival benefit in UCT in our cohort, as there was no difference between cell doses for survivors and those who died (median 4.7 × 10^7^/kg vs. 3.9 × 10^7^/kg, *P* = NS).

Our HSCT outcomes for ALL compare favorably with national and international studies [12,16,22,37–41]. Despite a significant shift towards alternate donor HSCT, overall engraftment times and length of initial hospital stay were unchanged. There was a significant increase in bacterial and viral (non-CMV) infections post-HSCT over time, but a decrease in IP. Discharge criteria remained the same over time. This suggests we are managing treatment-related toxicities with well-directed supportive care strategies. The increased incidence of bacterial and viral (non-CMV) infections probably reflects increased use of immunosuppressive therapies in Period 3, and improved testing modalities. CMV, PJP, and fungal infection incidence is unchanged over time, possibly reflecting disease prophylaxis in Period 3.

Our data show that contemporary HSCT outcomes are significantly improved and that outcomes for patients undergoing MSD and UCT are equivalent. This is consistent with previous literature [13]. Retrospective studies showed equivalent outcomes for MUD and UCT [35,42]. One recent study suggested MSD might be superior to alternate donor HSCT; however UCT data were not analyzed [21].

We postulate that improved OS by way of reduced TRM is due to multiple factors. These are likely to include selection of better matched UCT units with higher TNC doses [43] resulting in faster neutrophil engraftment times, a shorter time to transplant [27], better supportive care practices, availability of broader antimicrobials, improved prevention of and monitoring for CMV infection [44] and aggregate experience caring for HSCT patients [26]. Improvements in HSCT care have resulted in survival benefits for contemporary patients undergoing HSCT for high-risk ALL [45]. Units subjected to external audit have improved post-HSCT outcomes [46]. We expected to see improvement in MUD outcomes, due to introduction of high-resolution typing [3,14,28,47], but small numbers may have limited analysis.

The significant increase in patients transplanted in CR1 in Period 3 is not responsible for change in survival and TRM outcomes, as these significant improvements were also observed for patients transplanted in ≥CR2. Pre-HSCT status of patients transplanted in ≥CR2 was probably similar over time, as their median time to HSCT was unchanged over time.

Published studies, not confined to ALL in children, show reduced TRM over time [17,18,26,27]. Results are conflicting regarding TRM trends over time in pediatric ALL HSCT. Two publications reported significant improvement in OS and reduced TRM [16,22]. Another found no difference over time for OS post-unrelated HSCT, but did not discuss TRM trends for year of transplant [25].

This study was a single-center, retrospective analysis, with relatively small patient numbers. A strength of the study is uniform transplant practice since 2004, which represents the majority of Period 3. There was considerable heterogeneity in transplant practices in Periods 1 and 2. Uniform reporting of pre-HSCT performance scores would have provided additional comparison, although a surrogate marker, such as time from diagnosis to HSCT, has shown no change over time for patients transplanted in ≥CR2.

The incidence of aGVHD (Grades 2–4) was equivalent to retrospective series that included pediatric ALL [22,40,48]. Rates of aGHVD (Grade 3–4; 19.9%) were consistent with one prospective UCT study [43], but higher than another of MSD and MUD HSCT [3]. The higher proportion of UCT in our study likely contributed to this result. Higher aGVHD rates in UCT, as compared to other donor sources in our series, did not increase TRM or reduce relapse rates.

Post-HSCT relapse rates in our study, consistent with published ranges (18–37%) [3] were stable. Median follow-up times, including for Period 3 (median 45 months) were sufficient for detection of most relapses [20,27]. Although there was significant increase in TBI use in Period 3, this did not result in improved leukemic control, as rates of relapse were unchanged. Some pediatric ALL studies demonstrate reduction in post-HSCT relapse [27], while others do not [16,20,22,25,49]. The impact of increased numbers of patients in CR1 being transplanted on relapse rates is unclear. Taken together, this indicates that strategies to reduce post-HSCT relapse remain a priority.

Further studies using MRD may help determine extent of pre-HSCT cytoreduction [16], and help assess if contemporary patients in CR2 have more treatment-resistant disease [16,25] or increased risk of post-HSCT relapse [20,49]. Pre-transplant MRD >10^−3^ and disease status at transplant are the strongest predictors of relapse [4–8,13]. Additional factors may lead to increased relapse [42,43,50], including novel single nucleotide polymorphisms [51] and CMV D−/R− status [48].

Discovery of new cytoreductive agents [52,53] and establishing therapeutic pathways for post-HSCT MRD monitoring [4] are important. Prospective randomized pediatric HSCT studies need to compare UCT with both MSD and MUD outcomes, and HSCT versus chemotherapy-alone regimens. Validated pre-HSCT algorithms that incorporate performance scores and estimate TRM are required to assist in physician decision-making [17,25,54].

We found that contemporary outcomes for HSCT for pediatric ALL are significantly improved, due to several mechanisms, despite increased use of unrelated donors. TRM decreased significantly over 25 years. Relapse rates are static. International collaboration is required to target and develop novel treatment strategies for patients at high risk of relapse post-HSCT.

## Supporting Information

Figure S1. Survival over 25 years stratified for remission status.
