# Supplementary figures and images for: Transplant-Related Mortality Following Allogeneic Hematopoeitic Stem Cell Transplantation for Pediatric Acute Lymphoblastic Leukemia: 25-Year Retrospective Review
Source: Pediatr Blood Cancer. 2013 Jun 3;60(9):1520–7. doi: 10.1002/pbc.24559 (PMC3798104; doi:10.1002/pbc.24559)

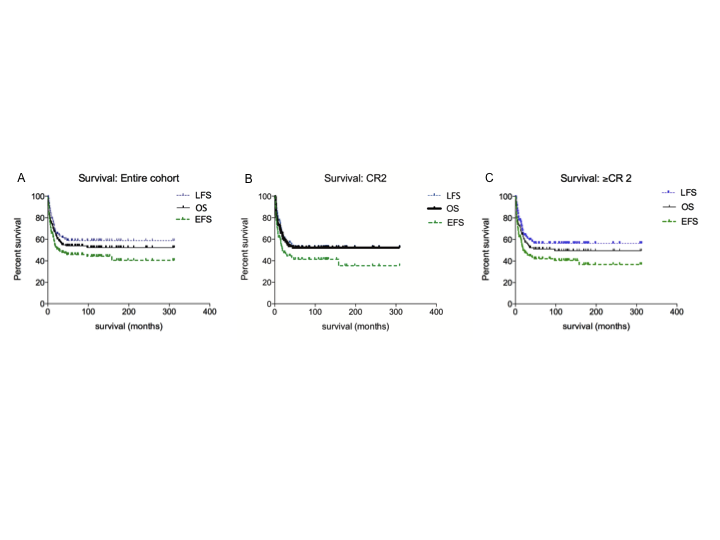

Supplement: Figure S1. — Survival over 25 years stratified for remission status. [file pbc0060-1520-sd1.tif]

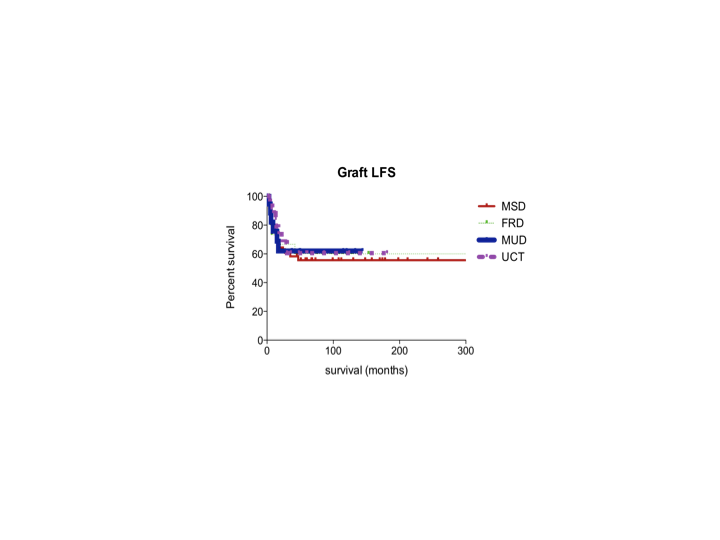

Supplement: Supplementary file 2 [file pbc0060-1520-sd2.tif]
